# Supplementary material for: Accelerometer-measured 24-hour movement behaviours over 7 days in Malaysian children and adolescents: A cross-sectional study
Source: PLoS One. 2024 Feb 20;19(2):e0297102. doi: 10.1371/journal.pone.0297102 (PMC10878504; doi:10.1371/journal.pone.0297102)
Supplement: S1 Table — (DOCX) [file pone.0297102.s001.docx]

**Supplementary Table S1:** Missing data

| **Variable** | | **Number missing** | **Percentage missing (%)** |
| --- | --- | --- | --- |
| *Age* | | 0 | 0 |
| *Sex* | | 0 | 0 |
| *Ethnicity* | | 0 | 0 |
| *Height* | | 1 | 0.16 |
| *Weight* | | 1 | 0.16 |
| *BMI z-score 2022* | | 1 | 0.16 |
| *BMI category 2022* | | 1 | 0.16 |
| *BMI z-score 2018* | | 4 | 0.64 |
| *BMI category 2018* | | 4 | 0.64 |
| *Change in BMI z-score* | | 5 | 0.80 |
| *Annual obesity incidence* | | 5 | 0.80 |
| *Highest education level in household* | | 2 | 0.32 |
| *Household income 2018* | | 10 | 1.60 |
| *Accelerometer PA:* | |  |  |
|  | *Sleep (mins/day)* | 135 | 21.57 |
|  | *Inactive time (mins/day)* | 135 | 21.57 |
|  | *LPA (mins/day)* | 135 | 21.57 |
|  | *MPA (mins/day)* | 135 | 21.57 |
|  | *VPA (mins/day)* | 135 | 21.57 |
|  | *MVPA (mins/day)* | 135 | 21.57 |
|  | *Intensity Gradient* | 136 | 21.73 |
|  | *Average Acceleration (mg)* | 135 | 21.57 |
| *AX6 Accelerometer PA (% of day)* | | 135 | 21.57 |
| *PAQ-C Score* | | 98 | 15.65 |
| *PAQ-C Domains:* | |  |  |
|  | *Organised/structured PA* | 35 | 5.59 |
|  | *Physical Education related PA* | 4 | 0.64 |
|  | *School recreational PA* | 26 | 4.15 |
|  | *Outside school PA* | 23 | 3.67 |
|  | *Weekend PA* | 16 | 2.56 |

Note: PA= physical activity, LPA= light intensity physical activity, MVPA= moderate to vigorous intensity physical activity, MPA= moderate physical activity, VPA= vigorous physical activity, BMI= body mass index.
