# Supplementary material for: Accelerometer-measured 24-hour movement behaviours over 7 days in Malaysian children and adolescents: A cross-sectional study
Source: PLoS One. 2024 Feb 20;19(2):e0297102. doi: 10.1371/journal.pone.0297102 (PMC10878504; doi:10.1371/journal.pone.0297102)
Supplement: S2 Table — (DOCX) [file pone.0297102.s002.docx]

**Supplementary Table S2:** Accelerometer-measured physical activity proportions (%/day) split by demographic characteristics

|  | | **Sleep duration (%/day)** | | **Inactive time (%/day)** | | **LPA (%/day)** | | **MVPA (%/day)** | | **MPA (%/day)** | | **VPA (%/day)** | |
| --- | --- | --- | --- | --- | --- | --- | --- | --- | --- | --- | --- | --- | --- |
|  | | **mean** | **95% CI** | **mean** | **95% CI** | **mean** | **95% CI** | **mean** | **95% CI** | **mean** | **95% CI** | **mean** | **95% CI** |
| ***Total*** | | 34.02 | 33.11-34.93 | 51.87 | 50.98-52.76 | 11.82 | 11.43-12.20 | 2.30 | 2.15-2.44 | 2.11 | 1.98-2.24 | 0.19 | 0.16-0.21 |
| ***Sex*** | |  |  |  |  |  |  |  |  |  |  |  |  |
|  | *Male* | 34.57 | 33.25-35.89 | 50.77 | 49.46-52.07 | 11.91 | 11.36-12.46 | 2.74 | 2.53-2.96 | 2.49 | 2.30-2.68 | 0.26 | 0.22-0.30 |
|  | *Female* | 33.48 | 32.26-34.73 | 52.94 | 51.73-54.15 | 11.72 | 11.18-12.26 | 1.86 | 1.68-2.04 | 1.74 | 1.58-1.90 | 0.12 | 0.10-0.14 |
| ***Age*** | |  |  |  |  |  |  |  |  |  |  |  |  |
|  | *Child* | 34.11 | 32.99-35.22 | 49.61 | 48.50-50.71 | 13.23 | 12.75-13.71 | 3.06 | 2.85-3.27 | 2.78 | 2.59-2.97 | 0.28 | 0.24-0.32 |
|  | *Adolescent* | 33.95 | 32.55-35.34 | 53.77 | 52.45-55.08 | 10.63 | 10.09-11.18 | 1.65 | 1.49-1.82 | 1.55 | 1.40-1.69 | 0.11 | 0.08-0.13 |
| ***Ethnicity*** | |  |  |  |  |  |  |  |  |  |  |  |  |
|  | *Malay* | 34.13 | 33.08-35.19 | 52.41 | 51.38-53.44 | 11.34 | 10.88-11.81 | 2.11 | 1.94-2.27 | 1.95 | 1.80-2.09 | 0.16 | 0.14-0.19 |
|  | *Chinese* | 32.57 | 30.69-34.46 | 52.94 | 51.03-54.86 | 11.95 | 11.14-12.76 | 2.54 | 2.18-2.89 | 2.30 | 2.00-2.60 | 0.24 | 0.17-0.31 |
|  | *Indian* | 35.43 | 32.02-38.85 | 47.35 | 44.28-50.42 | 14.23 | 13.15-15.32 | 2.98 | 2.51-3.45 | 2.74 | 2.32-3.16 | 0.24 | 0.17-0.30 |
| ***BMI Category*** | |  |  |  |  |  |  |  |  |  |  |  |  |
|  | *Underweight* | 35.60 | 32.74-38.47 | 50.70 | 46.34-55.06 | 11.23 | 8.90-13.55 | 2.47 | 1.48-3.47 | 2.28 | 1.40-3.15 | 0.20 | 0.07-0.33 |
|  | *Healthy weight* | 34.18 | 33.00-35.37 | 52.07 | 50.91-53.24 | 11.61 | 11.07-12.15 | 2.13 | 1.94-2.33 | 1.95 | 1.78-2.12 | 0.18 | 0.15-0.21 |
|  | *Overweight* | 33.57 | 31.44-35.70 | 51.54 | 49.42-53.66 | 12.39 | 11.51-13.26 | 2.51 | 2.18-2.84 | 2.31 | 2.02-2.60 | 0.20 | 0.15-0.25 |
|  | *Obese* | 33.77 | 31.56-35.98 | 51.82 | 49.75-53.89 | 11.93 | 11.19-12.66 | 2.49 | 2.18-2.79 | 2.30 | 2.03-2.57 | 0.19 | 0.15-0.23 |
| ***Highest education level in household*** | |  |  |  |  |  |  |  |  |  |  |  |  |
|  | *Up to Secondary* | 33.75 | 32.67-34.83 | 52.28 | 51.23-53.33 | 11.74 | 11.27-12.21 | 2.23 | 2.07-2.40 | 2.06 | 1.91-2.21 | 0.18 | 0.15-0.20 |
|  | *Tertiary* | 34.84 | 33.16-36.52 | 50.61 | 48.93-52.29 | 12.07 | 11.42-12.72 | 2.48 | 2.19-2.77 | 2.26 | 2.01-2.51 | 0.22 | 0.17-0.27 |
| ***Monthly household income 2018 (MYR)*** | |  |  |  |  |  |  |  |  |  |  |  |  |
|  | *<2000* | 33.61 | 32.29-34.93 | 52.50 | 51.25-53.76 | 11.72 | 11.18-12.25 | 2.17 | 1.97-2.37 | 2.00 | 1.83-2.18 | 0.17 | 0.14-0.20 |
|  | *≥2000* | 34.59 | 33.35-35.83 | 51.01 | 49.73-52.28 | 11.94 | 11.37-12.50 | 2.47 | 2.25-2.68 | 2.25 | 2.06-2.44 | 0.21 | 0.18-0.25 |

Note: LPA= light intensity physical activity, MVPA= moderate to vigorous intensity physical activity, MPA= moderate physical activity, VPA= vigorous physical activity, BMI= body mass index, CI= confidence interval, MYR= Ringgit Malaysia
