# Supplementary material for: Accelerometer-measured 24-hour movement behaviours over 7 days in Malaysian children and adolescents: A cross-sectional study
Source: PLoS One. 2024 Feb 20;19(2):e0297102. doi: 10.1371/journal.pone.0297102 (PMC10878504; doi:10.1371/journal.pone.0297102)
Supplement: S3 Table — (DOCX) [file pone.0297102.s003.docx]

**Supplementary Table S3:** Participants meeting WHO physical activity guidelines

|  | | **N (%) meeting MVPA guidelines** | **N (%) NOT meeting MVPA guidelines** |
| --- | --- | --- | --- |
| ***Overall*** | | 64 (13.0) | 427 (87.0) |
| ***Sex*** | |  |  |
|  | *Male* | 47 (19.4) | 195 (80.6) |
|  | *Female* | 17 (6.8) | 232 (93.2) |
| ***Age*** | |  |  |
|  | *Child* | 50 (22.3) | 174 (77.7) |
|  | *Adolescent* | 14 (5.2) | 253 (94.8) |
| ***Ethnicity*** | |  |  |
|  | *Malay* | 36 (10.6) | 305 (89.4) |
|  | *Chinese* | 15 (17.1) | 73 (83.0) |
|  | *Indian* | 13 (21.0) | 49 (79.0) |
| ***BMI Category*** | |  |  |
|  | *Underweight* | 3 (17.7) | 14 (82.4) |
|  | *Healthy weight* | 31 (11.2) | 245 (88.8) |
|  | *Overweight* | 14 (15.9) | 74 (84.1) |
|  | *Obese* | 15 (13.8) | 94 (86.2) |
| ***Highest education level in household*** | |  |  |
|  | *Up to Secondary* | 45 (12.5) | 316 (87.5) |
|  | *Tertiary* | 19 (14.7) | 110 (85.3) |
| ***Monthly household income 2018 (MYR)*** | |  |  |
|  | *<2000* | 29 (10.7) | 241 (89.3) |
|  | *≥2000* | 35 (16.2) | 181 (83.8) |

Note: MVPA= moderate-to-vigorous physical activity, BMI= body mass index, MYR= Ringgit Malaysia
