# Supplementary material for: Accelerometer-measured 24-hour movement behaviours over 7 days in Malaysian children and adolescents: A cross-sectional study
Source: PLoS One. 2024 Feb 20;19(2):e0297102. doi: 10.1371/journal.pone.0297102 (PMC10878504; doi:10.1371/journal.pone.0297102)
Supplement: S4 Table — (DOCX) [file pone.0297102.s004.docx]

**Supplementary Table S4:** Accelerometer-measured physical activity intensity gradient and average acceleration split by demographic characteristic

|  | | **Number of participants** | **Intensity Gradient** | | **Average Acceleration** | |
| --- | --- | --- | --- | --- | --- | --- |
|  | |  | **mean** | **95% CI** | **mean** | **95% CI** |
| ***Total*** | | 491 | -2.27 | -2.29 to -2.25 | 39.20 | 37.62-40.79 |
| ***Sex*** | |  |  |  |  |  |
|  | *Male* | 242 | -2.19 | -2.22 to -2.16 | 43.11 | 40.58-45.63 |
|  | *Female* | 248/249 | -2.35 | -2.37 to -2.32 | 35.41 | 33.57-37.25 |
| ***Age*** | |  |  |  |  |  |
|  | *Child* | 224 | -2.17 | -2.20 to -2.15 | 46.84 | 44.75-48.94 |
|  | *Adolescent* | 266/267 | -2.35 | -2.38 to -2.32 | 32.80 | 30.75-34.84 |
| ***Ethnicity*** | |  |  |  |  |  |
|  | *Malay* | 341 | -2.29 | -2.31 to -2.26 | 37.17 | 35.29-39.05 |
|  | *Chinese* | 88 | -2.22 | -2.27 to -2.18 | 41.06 | 37.44-44.67 |
|  | *Indian* | 61/62 | -2.23 | -2.29 to -2.18 | 47.76 | 43.20-52.32 |
| ***BMI Category*** | |  |  |  |  |  |
|  | *Underweight* | 17 | -2.27 | -2.37 to -2.16 | 40.06 | 29.03-51.10 |
|  | *Healthy weight* | 276 | -2.29 | -2.32 to -2.26 | 38.13 | 35.90-40.37 |
|  | *Overweight* | 88 | -2.24 | -2.29 to -2.20 | 41.60 | 38.12-45.07 |
|  | *Obese* | 108/109 | -2.24 | -2.28 to -2.20 | 39.66 | 36.67-42.66 |
| ***Highest education level in household*** | |  |  |  |  |  |
|  | *Up to Secondary* | 360/361 | -2.29 | -2.31 to -2.26 | 38.31 | 36.59-40.04 |
|  | *Tertiary* | 129 | -2.22 | -2.26 to -2.19 | 41.85 | 38.23-45.47 |
| ***Monthly household income 2018 (MYR)*** | |  |  |  |  |  |
|  | *<2000* | 269/270 | -2.30 | -2.33 to -2.27 | 37.78 | 35.56-40.00 |
|  | *≥2000* | 216 | -2.24 | -2.26 to -2.21 | 41.07 | 38.78-43.37 |

Note: BMI= body mass index, CI= confidence interval, MYR= Ringgit Malaysia
