# Supplementary material for: Accelerometer-measured 24-hour movement behaviours over 7 days in Malaysian children and adolescents: A cross-sectional study
Source: PLoS One. 2024 Feb 20;19(2):e0297102. doi: 10.1371/journal.pone.0297102 (PMC10878504; doi:10.1371/journal.pone.0297102)
Supplement: S5 Table — (DOCX) [file pone.0297102.s005.docx]

**Supplementary Table S5:** Regression models investigating differences in physical activity outcomes between demographic groups

|  | | **β** | **SE** | **t** | **p-value** | **β 95% CI** |
| --- | --- | --- | --- | --- | --- | --- |
| ***MVPA (min/day)*** | |  |  |  |  |  |
|  | *Sex- Female^a^* | -12.75* | 2.05 | -6.23 | 0.00 | -16.77 to -8.73 |
|  | *Age group- Adolescent^b^* | -20.23* | 1.93 | -10.49 | 0.00 | -24.02 to -16.44 |
|  | *Ethnicity- Indian^c^* | 12.57* | 3.20 | 3.93 | 0.00 | 6.29 to 18.85 |
|  | *Ethnicity- Chinese^c^* | 6.17* | 2.77 | 2.23 | 0.03 | 0.73 to 11.61 |
|  | *BMI category- underweight^d^* | 4.93 | 5.85 | 0.84 | 0.40 | -6.57 to 16.43 |
|  | *BMI category- overweight^d^* | 5.42 | 2.87 | 1.89 | 0.06 | -0.22 to 11.05 |
|  | *BMI category- obese^d^* | 5.09 | 2.65 | 1.92 | 0.06 | -0.12 to 10.30 |
|  | *Highest education level in household- tertiary^e^* | 3.57 | 2.41 | 1.48 | 0.14 | -1.16 to 8.31 |
|  | *Household income- ≥2000MYR/month^f^* | 4.25* | 2.15 | 1.98 | 0.049 | 0.02 to 8.48 |
| ***LPA (min/day)*** | |  |  |  |  |  |
|  | *Sex- Female^a^* | -2.73 | 5.63 | -0.49 | 0.63 | -13.79 to 8.32 |
|  | *Age group- Adolescent^b^* | -37.36* | 5.39 | -6.93 | 0.00 | -47.96 to -26.77 |
|  | *Ethnicity- Indian^c^* | 41.63* | 8.41 | 4.95 | 0.00 | 25.11 to 58.16 |
|  | *Ethnicity- Chinese^c^* | 8.70 | 7.28 | 1.19 | 0.23 | -5.61 to 23.01 |
|  | *BMI category- underweight^d^* | -5.48 | 15.57 | -0.35 | 0.73 | -36.07 to 25.11 |
|  | *BMI category- overweight^d^* | 11.20 | 7.63 | 1.47 | 0.14 | -3.79 to 26.18 |
|  | *BMI category- obese^d^* | 4.58 | 7.05 | 0.65 | 0.52 | -9.27 to 18.42 |
|  | *Highest education level in household- tertiary^e^* | 4.67 | 6.39 | 0.73 | 0.47 | -7.88 to 17.23 |
|  | *Household income- ≥200MYR/month^f^* | 3.17 | 5.71 | 0.56 | 0.58 | -8.04 to 14.39 |
| ***Inactive Time (min/day)*** | |  |  |  |  |  |
|  | *Sex- Female^a^* | 31.26* | 13.00 | 2.40 | 0.02 | 5.71 to 56.80 |
|  | *Age group- Adolescent^b^* | 59.93* | 12.84 | 4.67 | 0.00 | 34.70 to 85.17 |
|  | *Ethnicity- Indian^c^* | -72.64* | 19.72 | -3.68 | 0.00 | -111.40 to -33.89 |
|  | *Ethnicity- Chinese^c^* | 7.87 | 17.08 | 0.46 | 0.65 | -25.69 to 41.44 |
|  | *BMI category- underweight^d^* | -19.65 | 36.30 | -0.54 | 0.59 | -90.97 to 51.66 |
|  | *BMI category- overweight^d^* | -7.70 | 17.78 | -0.43 | 0.67 | -42.63 to 27.24 |
|  | *BMI category- obese^d^* | -3.66 | 16.43 | -0.22 | 0.82 | -35.94 to 28.63 |
|  | *Highest education level in household- tertiary^e^* | -24.00 | 14.81 | -1.62 | 0.11 | -53.09 to 5.09 |
|  | *Household income- ≥200MYR/month^f^* | -21.45 | 13.23 | -1.62 | 0.11 | -47.45 to 4.55 |
| ***Sleep (min/day)*** | |  |  |  |  |  |
|  | *Sex- Female^a^* | -15.80 | 13.30 | -1.19 | 0.24 | -41.93 to 10.33 |
|  | *Age group- Adolescent^b^* | -2.33 | 13.37 | -0.17 | 0.86 | -28.60 to 23.94 |
|  | *Ethnicity- Indian^c^* | 18.86 | 20.33 | 0.93 | 0.35 | -21.08 to 58.81 |
|  | *Ethnicity- Chinese^c^* | -22.32 | 17.61 | -1.27 | 0.21 | -56.91 to 12.27 |
|  | *BMI category- underweight^d^* | 20.53 | 36.95 | 0.56 | 0.58 | -52.06 to 93.13 |
|  | *BMI category- overweight^d^* | -8.81 | 18.10 | -0.49 | 0.63 | -44.38 to 26.75 |
|  | *BMI category- obese^d^* | -5.97 | 16.73 | -0.36 | 0.72 | -38.83 to 26.89 |
|  | *Highest education level in household- tertiary^e^* | 15.74 | 15.12 | 1.04 | 0.30 | -13.97 to 45.46 |
|  | *Household income- ≥2000MYR/month^f^* | 14.28 | 13.50 | 1.06 | 0.29 | -12.26 to 40.81 |

Note: Results of univariate linear regression models with the PA measures (MVPA/LPA/inactive time/sleep) as the dependent variables and the demographic variable as the independent variable. Statistical information about each model is presented by the p-value, with β representing the standardised beta coefficient of each predictor external event/factor. *= β indicating statistically significant difference between groups based on 95% confidence intervals. ^a^ = Compared to reference category ‘Sex- Male’, ^b^ = Compared to reference category ‘Age group- Child (7-12 years)’, ^c^ = Compared to reference category ‘Ethnicity- Malay’, ^d^ = Compared to reference category ‘BMI category- healthy weight’, ^e^ = Compared to reference category ‘Highest education level in household- Up to secondary education’, ^f^ = Compared to reference category ‘Household income- <200MYR/month’.

MVPA= moderate to vigorous intensity physical activity, LPA= light intensity physical activity, BMI= body mass index, CI= confidence interval, MYR= Ringgit Malaysia, SE= standard error.
