# Supplementary material for: Accelerometer-measured 24-hour movement behaviours over 7 days in Malaysian children and adolescents: A cross-sectional study
Source: PLoS One. 2024 Feb 20;19(2):e0297102. doi: 10.1371/journal.pone.0297102 (PMC10878504; doi:10.1371/journal.pone.0297102)
Supplement: S6 Table — (DOCX) [file pone.0297102.s006.docx]

**Supplementary Table S6:** Sensitivity analysis of regression models investigating differences in physical activity outcomes between demographic groups removing participants with data collection during Ramadan (3rd April – 1st May 2022)

|  | | **β** | **SE** | **t** | **p-value** | **β 95% CI** |
| --- | --- | --- | --- | --- | --- | --- |
| ***MVPA (min/day)*** | |  |  |  |  |  |
|  | *Ethnicity- India^a^* | 12.13* | 3.41 | 3.56 | 0.00 | 5.43 to 18.82 |
|  | *Ethnicity- Chinese^a^* | 4.45 | 2.93 | 1.52 | 0.13 | -1.32 to 10.22 |
| ***LPA (min/day)*** | |  |  |  |  |  |
|  | *Ethnicity- Indian^a^* | 45.05* | 8.82 | 5.11 | 0.00 | 27.72 to 62.39 |
|  | *Ethnicity- Chinese^a^* | 10.02 | 7.60 | 1.32 | 0.19 | -4.92 to 24.97 |
| ***Inactive Time (min/day)*** | |  |  |  |  |  |
|  | *Ethnicity- Indian^a^* | -73.10* | 19.44 | -3.76 | 0.00 | -111.31 to -34.89 |
|  | *Ethnicity- Chinese^a^* | 12.91 | 16.75 | 0.77 | 0.44 | -20.02 to 45.84 |
| ***Sleep (min/day)*** | |  |  |  |  |  |
|  | *Ethnicity- Indian^a^* | 16.46 | 19.79 | 0.83 | 0.41 | -22.45 to 55.36 |
|  | *Ethnicity- Chinese^a^* | -26.84 | 17.06 | -1.57 | 0.12 | -60.37 to 6.69 |

Note: Results of univariate linear regression models with the PA measures (MVPA/LPA/inactive time/sleep) as the dependent variables and ethnicity as the independent variable, including only participants with data collection outside of Ramadan (n=413). Statistical information about each model is presented by the p-value, with β representing the standardised beta coefficient of each predictor external event/factor. *= β indicating statistically significant difference between groups based on 95% confidence intervals. ^a^ = Compared to reference category ‘Ethnicity- Malay’.

MVPA= moderate to vigorous intensity physical activity, LPA= light intensity physical activity, SE= standard error.
