# Supplementary material for: Accelerometer-measured 24-hour movement behaviours over 7 days in Malaysian children and adolescents: A cross-sectional study
Source: PLoS One. 2024 Feb 20;19(2):e0297102. doi: 10.1371/journal.pone.0297102 (PMC10878504; doi:10.1371/journal.pone.0297102)
Supplement: S7 Table — (DOCX) [file pone.0297102.s007.docx]

**Supplementary Table S7:** Physical activity levels of participants with data collection during Ramadan (3rd April – 1st May 2022)

|  | | **Ramadan** | | | **Non-Ramadan** | | |
| --- | --- | --- | --- | --- | --- | --- | --- |
|  | | **n** | **mean** | **95% CI** | **n** | **mean** | **95% CI** |
| **Accelerometer-measured PA** | |  |  |  |  |  |  |
|  | *Sleep duration (min/day)* | 62 | 461.8 | 413.6-510.1 | 413 | 494.2 | 480.8-507.5 |
|  | *Inactive time (min/day)* | 62 | 778.4 | 733.1-823.7 | 413 | 740.6 | 727.3-753.9 |
|  | *LPA (min/day)* | 62 | 172.3 | 157.0-187.6 | 413 | 170.4 | 164.3-176.6 |
|  | *MVPA (min/day)* | 62 | 27.5 | 22.3-32.7 | 413 | 34.4 | 32.1-36.8 |
|  | *MPA (min/day)* | 62 | 25.5 | 21.1-29.9 | 413 | 31.6 | 29.5-33.6 |
|  | *VPA (min/day)* | 62 | 2.0 | 1.1-3.0 | 413 | 2.9 | 2.5-3.2 |
| **PAQ-C measured PA** | |  |  |  |  |  |  |
|  | *PAQ-C score* | 54 | 1.97 | 1.82-2.13 | 458 | 2.23 | 2.17-2.29 |
|  | *Organised/structured PA* | 59 | 1.27 | 1.21-1.33 | 516 | 1.36 | 1.33-1.38 |
|  | *Physical Education related PA* | 62 | 1.79 | 1.53-2.05 | 544 | 2.46 | 2.34-2.57 |
|  | *School recreational PA* | 62 | 1.69 | 1.50-1.89 | 522 | 1.83 | 1.75-1.91 |
|  | *Outside school PA* | 60 | 2.40 | 2.10-2.70 | 527 | 2.65 | 2.55-2.75 |
|  | *Weekend PA* | 61 | 2.57 | 2.30-2.85 | 533 | 2.71 | 2.61-2.81 |

Note: LPA= light intensity physical activity, MVPA= moderate to vigorous intensity physical activity, PA= physical activity, MPA= moderate physical activity, VPA= vigorous physical activity, CI= confidence interval. The PAQ-C questionnaire is scored on a 5-point Likert scale, with a higher score indicating higher level of activity.
