# Supplementary material for: Accelerometer-measured 24-hour movement behaviours over 7 days in Malaysian children and adolescents: A cross-sectional study
Source: PLoS One. 2024 Feb 20;19(2):e0297102. doi: 10.1371/journal.pone.0297102 (PMC10878504; doi:10.1371/journal.pone.0297102)
Supplement: S8 Table — (DOCX) [file pone.0297102.s008.docx]

**Supplementary Table S8:** Physical activity levels of participants with data collection during COVID-19 related rotating school systems

|  | | **COVID-19 related rotating school systems** | | | **No COVID-19 related rotating school systems** | | |
| --- | --- | --- | --- | --- | --- | --- | --- |
|  | | **n** | **mean** | **95% CI** | **n** | **mean** | **95% CI** |
| **Accelerometer-measured PA** | |  |  |  |  |  |  |
|  | *Sleep duration (min/day)* | 254 | 492.4 | 476.2-508.5 | 206 | 490.1 | 467.1-513.1 |
|  | *Inactive time (min/day)* | 254 | 757.5 | 741.4-773.6 | 206 | 729.6 | 707.8-751.4 |
|  | *LPA (min/day)* | 254 | 159.2 | 151.4-166.9 | 206 | 183.3 | 174.9-191.7 |
|  | *MVPA (min/day)* | 254 | 30.4 | 27.7-33.1 | 206 | 37.0 | 33.6-40.4 |
|  | *MPA (min/day)* | 254 | 28.0 | 25.6-30.5 | 206 | 33.8 | 30.8-36.8 |
|  | *VPA (min/day)* | 254 | 2.4 | 1.9-2.8 | 206 | 3.1 | 2.6-3.7 |
| **PAQ-C measured PA** | |  |  |  |  |  |  |
|  | *PAQ-C score* | 210 | 2.18 | 2.09-2.27 | 292 | 2.24 | 2.16-2.32 |
|  | *Organised/structured PA* | 235 | 1.33 | 1.30-1.36 | 326 | 1.37 | 1.34-1.40 |
|  | *Physical Education related PA* | 251 | 1.80 | 1.67-1.93 | 340 | 2.86 | 2.71-3.00 |
|  | *School recreational PA* | 242 | 1.86 | 1.74-1.98 | 327 | 1.81 | 1.71-1.90 |
|  | *Outside school PA* | 242 | 2.68 | 2.53-2.82 | 332 | 2.61 | 2.49-2.74 |
|  | *Weekend PA* | 249 | 2.71 | 2.57-2.85 | 331 | 2.70 | 2.58-2.83 |

Note: LPA= light intensity physical activity, MVPA= moderate to vigorous intensity physical activity, PA= physical activity, MPA= moderate physical activity, VPA= vigorous physical activity, CI= confidence interval. The PAQ-C questionnaire is scored on a 5-point Likert scale, with a higher score indicating higher level of activity.
