# Supplementary material for: Accelerometer-measured 24-hour movement behaviours over 7 days in Malaysian children and adolescents: A cross-sectional study
Source: PLoS One. 2024 Feb 20;19(2):e0297102. doi: 10.1371/journal.pone.0297102 (PMC10878504; doi:10.1371/journal.pone.0297102)
Supplement: S9 Table — (DOCX) [file pone.0297102.s009.docx]

**Supplementary Table S9:** Linear regression model results for associations between external events with moderate-to-vigorous physical activity

|  | | **MVPA (min/day)** | | | | |
| --- | --- | --- | --- | --- | --- | --- |
|  | | **β** | **SE** | **t** | **p-value** | **β 95% CI** |
| ***Ramadan^a^*** | |  |  |  |  |  |
|  | *Model 1 (unadjusted)* | 6.93* | 3.21 | 2.16 | 0.03 | 0.62 to 13.24 |
|  | *Model 2a (adjusted for age and sex)* | 5.09 | 2.81 | 1.81 | 0.07 | -0.44 to 10.61 |
| ***COVID-19 related rotating schooling systems^b^*** | |  |  |  |  |  |
|  | *Model 1 (unadjusted)* | 6.57* | 2.20 | 2.99 | 0.003 | 2.26 to 10.88 |
|  | *Model 2b (adjusted for age, sex and ethnicity)* | 3.50 | 2.72 | 1.29 | 0.20 | -1.84 to 8.84 |

Note: Model 1: Simple linear regression models between each individual external event/factor (independent variable) with accelerometer-measured MVPA (dependent variable). Model 2a: Regressions included confounders (age, sex) as predictors for each indicator of mental health and wellbeing in separate analyses. Model 2b: Regressions included all confounders (age, sex, ethnicity) as predictors for each indicator of mental health and wellbeing in separate analyses. Statistical information about each model is presented by the p-value, with β representing the standardised beta coefficient of each predictor external event/factor.

MVPA= moderate to vigorous physical activity, CI= confidence interval, SE= standard error, ^a^= Compared to reference category ‘Data collection during Ramadan’, ^b^= Compared to reference category ‘Data collection during COVID-19 related rotating schooling systems’.
